# Automated Remote Monitoring of Depression: Acceptance Among Low-Income Patients in Diabetes Disease Management

**DOI:** 10.2196/mental.4823

**Published:** 2016-01-25

**Authors:** Magaly Ramirez, Shinyi Wu, Haomiao Jin, Kathleen Ell, Sandra Gross-Schulman, Laura Myerchin Sklaroff, Jeffrey Guterman

**Affiliations:** ^1^ Daniel J Epstein Department of Industrial and Systems Engineering University of Southern California Los Angeles, CA United States; ^2^ Edward R Roybal Institute on Aging School of Social Work University of Southern California Los Angeles, CA United States; ^3^ RAND Corporation Santa Monica, CA United States; ^4^ School of Social Work University of Southern California Los Angeles, CA United States; ^5^ Los Angeles County Department of Health Services Los Angeles, CA United States; ^6^ College of Behavioral and Social Sciences California State University, Northridge Northridge, CA United States; ^7^ David Geffen School of Medicine University of California, Los Angeles Los Angeles, CA United States

**Keywords:** technology assessment, telecommunications, telemedicine, patient care management, clinical decision support systems, depression, diabetes mellitus, safety-net clinics

## Abstract

**Background:**

Remote patient monitoring is increasingly integrated into health care delivery to expand access and increase effectiveness. Automation can add efficiency to remote monitoring, but patient acceptance of automated tools is critical for success. From 2010 to 2013, the Diabetes-Depression Care-management Adoption Trial (DCAT)–a quasi-experimental comparative effectiveness research trial aimed at accelerating the adoption of collaborative depression care in a safety-net health care system–tested a fully automated telephonic assessment (ATA) depression monitoring system serving low-income patients with diabetes.

**Objective:**

The aim of this study was to determine patient acceptance of ATA calls over time, and to identify factors predicting long-term patient acceptance of ATA calls.

**Methods:**

We conducted two analyses using data from the DCAT technology-facilitated care arm, in which for 12 months the ATA system periodically assessed depression symptoms, monitored treatment adherence, prompted self-care behaviors, and inquired about patients’ needs for provider contact. Patients received assessments at 6, 12, and 18 months using Likert-scale measures of willingness to use ATA calls, preferred mode of reach, perceived ease of use, usefulness, nonintrusiveness, privacy/security, and long-term usefulness. For the first analysis (patient acceptance over time), we computed descriptive statistics of these measures. In the second analysis (predictive factors), we collapsed patients into two groups: those reporting “high” versus “low” willingness to use ATA calls. To compare them, we used independent *t* tests for continuous variables and Pearson chi-square tests for categorical variables. Next, we jointly entered independent factors found to be significantly associated with 18-month willingness to use ATA calls at the univariate level into a logistic regression model with backward selection to identify predictive factors. We performed a final logistic regression model with the identified significant predictive factors and reported the odds ratio estimates and 95% confidence intervals.

**Results:**

At 6 and 12 months, respectively, 89.6% (69/77) and 63.7% (49/77) of patients “agreed” or “strongly agreed” that they would be willing to use ATA calls in the future. At 18 months, 51.0% (64/125) of patients perceived ATA calls as useful and 59.7% (46/77) were willing to use the technology. Moreover, in the first 6 months, most patients reported that ATA calls felt private/secure (75.9%, 82/108) and were easy to use (86.2%, 94/109), useful (65.1%, 71/109), and nonintrusive (87.2%, 95/109). Perceived usefulness, however, decreased to 54.1% (59/109) in the second 6 months of the trial. Factors predicting willingness to use ATA calls at the 18-month follow-up were perceived privacy/security and long-term perceived usefulness of ATA calls. No patient characteristics were significant predictors of long-term acceptance.

**Conclusions:**

In the short term, patients are generally accepting of ATA calls for depression monitoring, with ATA call design and the care management intervention being primary factors influencing patient acceptance. Acceptance over the long term requires that the system be perceived as private/secure, and that it be constantly useful for patients’ needs of awareness of feelings, self-care reminders, and connectivity with health care providers.

**Trial Registration:**

ClinicalTrials.gov NCT01781013; https://clinicaltrials.gov/ct2/show/NCT01781013 (Archived by WebCite at http://www.webcitation.org/6e7NGku56)

## Introduction

In late 2014, the Centers for Medicare and Medicaid Services (CMS) issued new rules that expanded provider reimbursements beginning in 2015 for remote monitoring of Medicare beneficiaries [1]. Telemedicine and telehealth—or, what Kvedar and colleagues refer to collectively as “connected health”—capitalize on advances in health information technology (HIT) to remotely provide health care services, information, health education, and self-management support [2]. A number of studies have demonstrated the potential of these technologies to increase access and quality of care while decreasing health care costs [3-8].

Given the mounting evidence for the clinical and cost effectiveness of connected health, the CMS ruling is likely to boost interest in its adoption. Attempts to improve patient care with connected health, however, will be futile unless patients accept these technologies. Prior studies suggest that individuals who do not accept technologies simply will not use them [9,10]. Indeed, so critical is user acceptance that it has been regarded as “the pivotal factor in determining the success or failure of an information system” [11]. In an editorial review of connected health technologies to support behavior changes for self-management, Piette [12] remarks that patients’ discontinued use, which results from a lack of acceptance, has largely hindered large-scale implementation. Therefore, it is clear that patient acceptance has important implications for the broader domain of connected health, since patients who do not accept (and thus do not use) these technologies will not realize the full benefits of them, resulting in a loss for both patients and payers.

This study investigates patient acceptance of an automated telecommunications system designed to facilitate depression care management of low-income patients with diabetes in a safety-net care system [13,14]. There is evidence of significant disparities in receipt of depression treatment in low-income, uninsured, and minority populations. These groups are less likely to receive depression care [15-19], show greater treatment discontinuation [20], and experience higher rates of clinically significant depression. Patient barriers to depression care influence detection and treatment processes. For example, minority patients are less likely to voluntarily report depressive symptoms, may view depression as a moral weakness or character flaw instead of an illness, may be more likely to ascribe symptoms of depression to a physical illness [21,22], and may refuse or discontinue treatment due to stigma [23]. Nonadherence to depression treatment in minority groups with diabetes is common, due in part to side effects of diabetes medications [24-26]. Further exacerbating the challenges are cost and complex patient-provider interactions inherent in caring for patients with comorbid chronic illnesses. For instance, prioritizing among competing demands may negatively affect initiation and long-term follow-up of depression management in primary care [27-34].

To address these issues in order to accelerate the adoption of evidence-based depression care [24,35], we designed an advanced automated telephonic assessment (ATA) system. It had the capability to inquire—via periodic telephone calls to patients—about important aspects of depression care using a combination of the following six modules: monitoring for depression, assessing pain, assessing adherence to antidepressant medications, assessing psychotherapy practice, prompting depression self-care activities, and allowing patients to request contact from a clinician [14]. The ATA system was fully integrated into an existing disease management registry (DMR), which allowed it to automatically select these modules and the frequency of calls depending on individual patient clinical data: results from previous ATA calls or clinical assessments (depressed patients were called monthly, nondepressed patients quarterly), whether patients had an active antidepressant medication prescription, and whether patients were receiving psychotherapy. It also allowed patients to indicate their preferences for language (English or Spanish), call days and times, and receiving human calls instead of machine calls. If a call was not answered, the ATA system attempted again three times per day for 7 days (morning, afternoon, and evening). As a whole, the design allowed the ATA system to individually customize calls to focus on patients’ specific needs and preferences rather than having patients adapt to standard comprehensive assessments, in essence illustrating the philosophy of patient-centered care.

Moreover, the ATA system facilitated timely, proactive follow-up by clinicians and staff. Data captured on the ATA calls were automatically assessed and the results sent to the DMR for clinician and staff review [14]. Notifications, tasks, and alerts were triggered in response to specific issues identified from the ATA calls: patient requests for contact, high depression scores, nonadherence to antidepressant medications, or suicidal ideation.

The ATA system was tested in the Diabetes-Depression Care-management Adoption Trial (DCAT) [13]. DCAT was a 12-month, quasi-experimental comparative effectiveness research trial conducted in collaboration with the Los Angeles County Department of Health Services (LACDHS) with the aim of comparing different approaches for accelerating the adoption of collaborative team depression care in routine safety-net primary care practice. The study was conducted in its ambulatory care clinics serving low-income, racially/ethnically diverse (but primarily Hispanic/Latino) patients. It tested three depression care delivery models: usual care (UC), supported care (SC), and technology-facilitated care (TC). UC represented the status quo, whereby primary care providers (PCPs) and their staff initiate the translation and adoption of depression care evidence. Both SC and TC included care teams of the LACDHS disease management program (DMP) for the first 6 months of the trial to support diabetes care as well as depression care using evidence-based protocols [36]. After 6 months, patients returned to their PCPs for care. The difference between SC and TC was that the latter utilized the ATA system for 12 months to facilitate automated depression screening and monitoring, and timely follow-up by clinicians and staff. The provider notifications, tasks, and alerts generated by the ATA system were sent to DMP teams during the first 6 months of DCAT and to PCPs and their staff during the second 6 months.

If such automated remote screening and monitoring of depression—and more broadly, connected health—is to be integrated into mainstream health care delivery to help reach the important policy goal of expanding access to high-quality, effective, and efficient care, an understanding of patient technology acceptance is urgently needed. Studies on remote assessment and monitoring via connected health, however, continue to overlook this important research area [35,37]. Those that do touch upon elements of patient acceptance tend to be cross-sectional and operationalize the construct using measures of patient satisfaction with care, which in itself reveals little about technology acceptance or how to design the system to improve patient acceptance.

The present study echoes the information technology literature [38-40] by measuring technology acceptance as patients’ willingness to use ATA calls as part of their depression care. Moreover, this study is longitudinal, which allows for an understanding of how patient acceptance may change over time. Finally, to inform future design choices for automated remote depression monitoring technology, the evaluation includes several system characteristics that may explain why patients accept or reject the technology. Thus, in sum, the study was undertaken to (1) determine patient acceptance of ATA calls for remote depression screening and monitoring over time, and (2) identify what factors predict long-term patient acceptance of ATA calls.

## Methods

### Study Design and Participants

To answer the research questions, we analyzed data collected from patients in the TC arm of DCAT. English-Spanish bilingual interviewers administered assessments of technology acceptance at 6, 12, and 18 months. Thus, patients received two assessments during the study and one assessment 6 months after the study had ended.

### Survey-Based Measures of ATA Call Acceptance

DCAT defined technology acceptance as patients’ reported willingness to use ATA calls in the future as part of their depression care. The measurement was administered at 6, 12, and 18 months. DCAT also assessed additional measures of ATA call design characteristics: perceived ease of use (7 items), perceived usefulness (6 items), perceived nonintrusiveness (3 items), and perceived privacy/security (1 item). DCAT administered these assessments at 6 and 12 months. Moreover, patients’ preference for mode of reach (1 item) was assessed at 6, 12, and 18 months. Finally, at 18 months, patients were asked about their long-term perceived usefulness of ATA calls (3 items). All measures were assessed on a 5-point Likert scale. Table 1 provides the exact wording used in the DCAT assessments.

**Table 1:** Measures of patient ATA call acceptance.

Domain of measurement	Items	Administration
Willingness to use ATA calls^a^	To what extent do you agree or disagree with the following statement?	6, 12, and 18 months
	You would not mind receiving automated calls as part of your depression care in the future.	
Perceived ease of use^b,c^	“How often would you say...”	6 and 12 months
	The language used by Amy^c^ in the calls was easy for you to understand?	
	Amy’s voice on the call was loud enough to hear without straining?	
	Amy was speaking too fast on the automated call?	
	You were clear on how to respond to Amy’s questions?	
	You had difficulty answering the questions when asked to press buttons on your phone?	
	Giving answers to a real person would have been easier than giving answers to the automated operator Amy?	
	Amy had difficulty understanding you when you responded verbally?	
Perceived usefulness^b,c^	“How often would you say...”	6 and 12 months
	The call made you feel confident that your nurse or social worker knew how you were doing?	
	The calls made you feel like your nurse of social worker was more accessible?	
	The calls by Amy were just as effectiveness in reporting your feelings as an in-person visit with your care provider?	
	The antidepressant medication questions asked by Amy reminded you to take your medications?	
	The problem-solving skills questions asked by Amy reminded you to use these skills?	
	The calls reminded you to do things like a physical activity or a fun activity?	
Perceived nonintrusiveness^b^	“How often would you say...”	6 and 12 months
	You enjoyed receiving the calls?	
	You felt the calls were a bother?	
	The length of the calls seemed about right?	
Perceived privacy/security^a^	To what extent do you agree or disagree with the following statement?	6 and 12 months
	You feel automated calls are private and/or secure.	
Preferred mode of reach	To what extent do you agree or disagree with the following statement?	6, 12, and 18 months
	Instead of receiving automated calls, you would prefer to call the automated service at your convenience.	
Long-term perceived usefulness^a^	To what extent do you agree or disagree with the following statements?	18 months
	The automated calls helped you be more aware of how you are feeling.	
	The automated calls reminded you to take care of your health, such as doing exercise.	
	The automated calls helped you stay better connected with your doctors, nurses or social worker.	

^a^Patients responded using a 5-point Likert scale of agreement (1=strongly disagree, 2=disagree, 3=neutral, 4=agree, and 5=strongly agree).

^b^Patients responded using a 5-point Likert scale of frequency (1=never, 2=rarely, 3=about half the time, 4=usually, and 5=always).

^c^“Amy” was the persona of the ATA calls

### ATA Call Completion Rates

We assessed the rate of completed ATA calls for three periods: 0-6 months, 7-12 months, and 0-12 months. An ATA call was defined as complete if it reached the patient and recorded answers to the depression assessment questions: PHQ-2 or PHQ-9, whichever was asked.

### Statistical Analysis

We conducted two analyses: one to determine patient acceptance of ATA calls for remote depression screening and monitoring over time, and the other to identify what factors predict long-term patient acceptance of ATA calls. Sample characteristics and sample sizes for each analysis are shown in the Results section (Table 2).

For the first analysis (patient acceptance over time), we included the DCAT TC arm patients who provided responses for a given survey-based measure at each of the measurement periods. By excluding patients who did not meet this criterion, we were able to estimate changes more accurately for each measure over time. We computed descriptive statistics of all measures. For those measures consisting of multiple items, we computed the average points across items and rounded the average to the nearest integer. Furthermore, we conducted a paired *t* test to determine if there was a significant difference between the ATA call completion rates from 0 to 6 months and from 6 to 12 months. We also used Spearman rank correlation to test the association between the ATA call completion rate of months 0 to 12 and the survey-based measures of ATA call acceptance.

In the second analysis (predictive factors), we used a different criterion to select patients from among the pool of TC arm patients: patients who responded to the question of willingness to use ATA calls at 18 months and at least once at 6 or 12 months or both. If patients answered the question at both 6 and 12 months, we computed the average for use in the analysis. The 125 patients in this sample were collapsed into two groups: those reporting “high” willingness to use ATA calls at 18 months (Likert scale response of 4 or higher) and those reporting “low” willingness to use ATA calls at 18 months (all other response categories). We compared the descriptive statistics for the two groups: patient sociodemographic characteristics, health conditions, health care utilization, and ATA call completion rate. We also compared their responses for perceived ease of use, perceived usefulness, perceived nonintrusiveness, perceived privacy/security, preference of ATA call mode, and long-term perceived usefulness. If patients completed assessments of these measures at both 6 and 12 months, we computed the average of the two for use in the analysis. To compare the two groups of patients, we used independent *t* tests for continuous variables and Pearson chi-square tests for categorical variables. Next, we jointly entered independent factors found to be significantly associated with 18-month willingness to use ATA calls at the univariate level into a logistic regression model with backward selection to identify predictive factors. Then, we performed a final logistic regression model with the identified significant predictive factors and reported the odds ratio estimates and 95% confidence intervals. All analyses were conducted at 0.05 significance level (2-tailed) using IBM SPSS software, version 22.0.

## Results

### Sample Characteristics

Table 2 provides the characteristics of patients in the two samples used in the two analyses. The majority of patients were female, Hispanic/Latino, and preferred Spanish as their primary language. The characteristics of the two samples were not significantly different from one another. A comparison of these samples with the rest of the patients in DCAT TC excluded from the analyses did reveal significant differences in characteristics (see Tables A-1 and A-2 in Multimedia Appendix 1). Compared to the rest of DCAT TC, the two samples had a greater proportion of Hispanics/Latinos, reported a higher willingness to use ATA calls at 6 and 12 months, and had a higher ATA call completion rate. The sample for the second analysis also had lower blood sugar values, better diabetes self-care, and reported higher perceived ease of use and perceived nonintrusiveness at 6 and 12 months compared to the rest of patients in the TC arm of DCAT.

**Table 2:** Patient characteristics for samples in the two analyses (no statistically significant difference between the two samples).

Characteristic	Sample for first analysis		Sample for second analysis	
	N	Statistics^a^	N	Statistics^a^
Female	109	72 (66.1%)	125	80 (64.0%)
Age	109	51.94 (9.01)	125	51.31 (8.81)
Hispanic/Latino	109	105 (96.3%)	125	116 (92.8%)
Spanish as preferred language	109	93 (85.0%)	125	104 (83.2%)
Married	109	49 (45.0%)	125	55 (44.0%)
PHQ-9 (range 0-27, higher=more severe depression)^b,c^	109	5.73 (4.93)	125	5.65 (4.60)
Total number of socioeconomic stressors^c^	109	2.28 (1.56)	125	2.37 (1.46)
SCL-20, mean score^c,d^	109	0.54 (0.53)	125	0.51 (0.48)
SF-12 mental (general population=50, higher=better)^c,e^	109	50.54 (9.15)	125	51.08 (9.03)
Time with diabetes in years	107	10.15 (7.42)	124	9.98 (7.05)
On insulin treatment^c^	109	82 (75.2%)	125	89 (71.2%)
BMI^c,f^	109	32.93 (6.55)	125	32.75 (6.16)
A1C value^c,g^	108	8.87 (1.39)	124	8.72 (1.39)
Low-density lipoprotein cholesterol^c^	108	167.08 (36.20)	124	168.44 (36.60)
Whitty-9 diabetes symptoms (range 1-5, 1=none to 5=every day)^c^	109	1.64 (0.54)	125	1.62 (0.49)
Number of diabetes complications^c^	109	1.26 (0.89)	125	1.22 (0.79)
Toolbert diabetes self-care in the past 7 days (range 0-7)^c^	109	4.63 (0.98)	125	4.65 (1.01)
Diabetes emotional burden (range 1-5, 1=not a problem to 5=very burdensome)^c^	109	2.53 (1.35)	125	2.48 (1.37)
Diabetes regime distress (range 1-5, 1=not a problem to 5=very burdensome)^c^	109	2.19 (1.14)	125	2.13 (1.17)
Self-rated health (range 1-5, 1=poor to 5=excellent)^c^	109	2.29 (0.60)	125	2.34 (0.60)
Chronic pain^c^	109	17 (15.6%)	125	24 (19.2%)
SF-12 physical (general population=50, higher=better health)^c,e^	109	43.18 (9.62)	125	43.17 (9.49)
Sheehan disability scale (range 0-10, 0=none to 10=extremely)^c^	109	2.21 (2.34)	125	2.14 (2.26)
Number of ICD-9 diagnosis^c,h^	108	8.60 (4.50)	124	8.46 (4.46)
Number of clinic visits^c^	107	10.44 (5.61)	124	10.56 (5.64)
Number of emergency room visits^c^	41	1.33 (0.61)	44	1.33 (0.60)
Number of hospitalizations^c^	15	1.47 (0.83)	18	1.39 (0.78)
Willingness to use^c^	109	4.02 (0.93)	125	4.00 (1.08)
Perceived ease of use^c^	109	4.05 (0.56)	125	4.12 (0.50)
Perceived usefulness^c^	109	3.63 (0.89)	125	3.69 (0.90)
Perceived nonintrusiveness^c^	109	4.20 (0.87)	125	4.29 (0.84)
Perceived privacy/security^c^	109	4.10 (1.11)	125	4.17 (1.08)
Preference of ATA call mode^c^	109	3.82 (1.06)	125	3.58 (1.32)
Long-term perceived usefulness	76	3.71 (0.92)	125	3.74 (0.99)
ATA call completion rate^c^	108	0.70 (0.26)	123	0.74 (0.24)

^a^Values are numbers (column percentages) for categorical variables and mean (SD) for continuous variables.

^b^Patient Health Questionnaire, 9 items

^c^Assessment at 6 or 12 months. If both were available, then the average was taken.

^d^Symptoms CheckList, 20 items

^e^Short-Form Health Survey, 12 items

^f^Body mass index

^g^Glycated hemoglobin test

^h^International Classification of Diseases, 9th revision

### First Analysis: Patient Acceptance of ATA Calls Over Time

**Figure 1:**
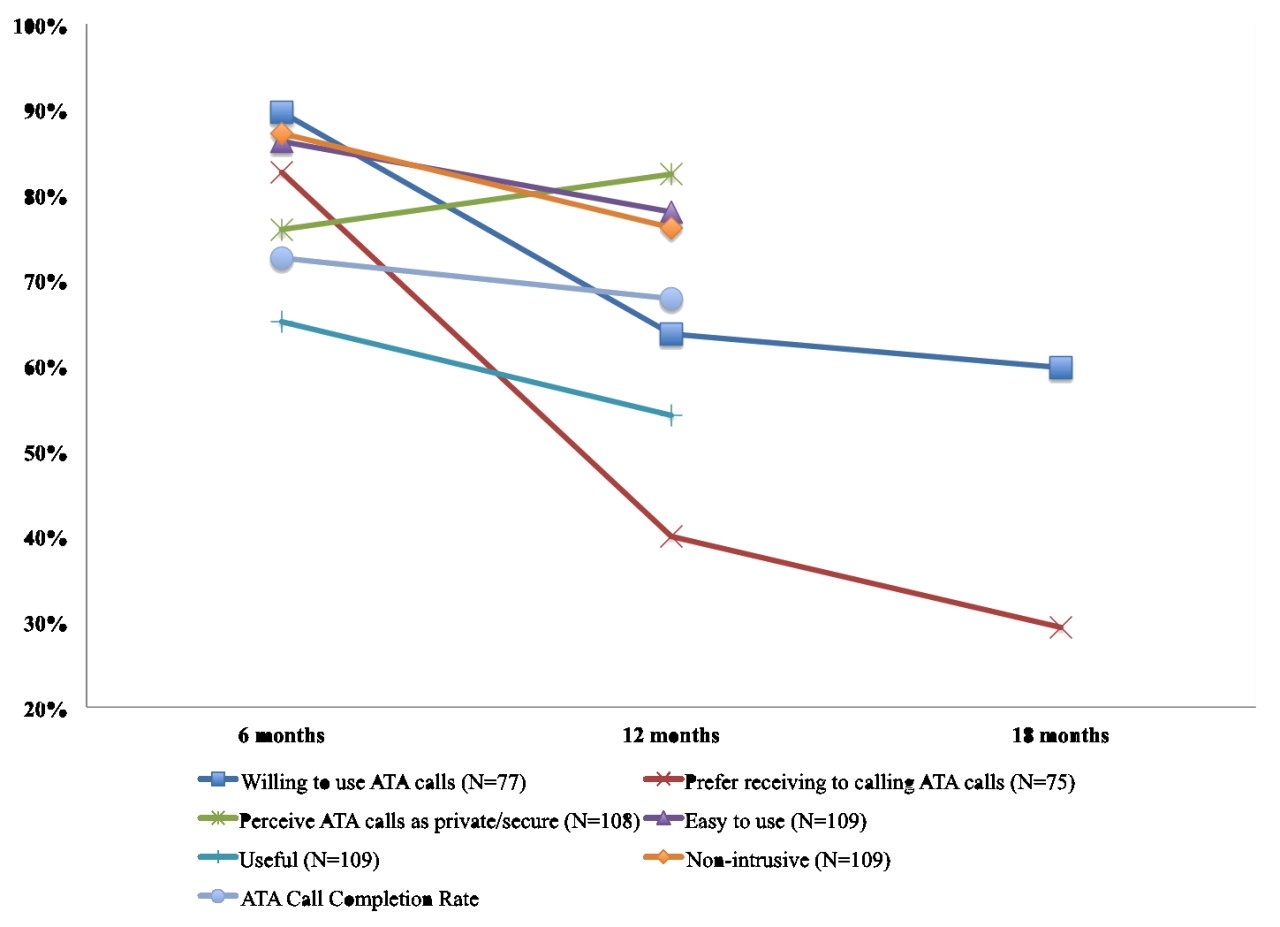
Patient acceptance of ATA calls over time.

Figure 1 illustrates patient acceptance of ATA calls over time. In the first 6 months of the trial, 90% (69/77) of patients reported a high willingness to use ATA calls. At 12 and 18 months, however, the proportion of patients reporting a high willingness to use ATA calls decreased to 64% (49/77) and 60% (46/77), respectively. After 6 months in the trial, 83% (62/75) of patients agreed or strongly agreed that they would prefer to receive automated calls rather than calling the ATA system at their convenience. The proportion of patients reporting this decreased to 40% (30/75) and 29% (22/75) at 12 and 18 months, respectively. Throughout the trial, most patients agreed or strongly agreed that ATA calls felt private/secure (82/108 at 6 months, 89/108 at 12 months). At 6 months, 86.2% (94/109) of patients reported that ATA calls were usually or always easy to use. This number decreased to 78.0% (85/109) at 12 months. The proportion of patients reporting that ATA calls were usually or always useful decreased from 65.1% (71/109) at 6 months to 54.1% (59/109) at 12 months. At the 18-month follow-up, 51.0% (64/125) of patients agreed or strongly agreed that the ATA calls were useful. At 6 months, most patients 87% (95/109) perceived that ATA calls were usually or always nonintrusive. More patients perceived the calls to be intrusive after 12 months in the trial as is evident from a decrease in the proportion of patients who reported otherwise 76% (83/109).

The ATA average call completion rate was 72.6% and 67.8% at 6 and 12 months, respectively—the difference between the two was not statistically significant (*P*=.10). In investigating the associations between the ATA call completion rate of months 0 to 12 (70.2%) and the various survey-based acceptance measures, only two measures were statistically significant: (1) perceived ease of use (Spearman correlation coefficient=0.25, *P*=.008) and (2) perceived nonintrusiveness (Spearman correlation coefficient=0.27, *P*=.004).

### Second Analysis: Factors Predicting Patient Acceptance of ATA Calls

We compared patients who reported, at 18 months, a high willingness to use ATA with patients reporting low willingness to use ATA calls to determine how the two groups differed in terms of the various sociodemographic characteristics, health conditions, health care utilization, and ATA-related measures listed in Table 2. Table 3 provides results for characteristics where there was a statistically significant difference between the two groups. See Table A-3 in Appendix for full results.

**Table 3:** Characteristics of patients reporting high versus low willingness to use ATA calls at 18 months.

Characteristic	High willingness to use ATA calls at 18 months		Low willingness to use ATA calls at 18 months		*P* ^b^
	N	Statistics^a^	N	Statistics^a^	
Toolbert diabetes self-care in the past 7 days (range 0-7)^c^	74	4.81 (0.95)	51	4.43 (1.05)	.03
Willingness to use^c^	74	4.17 (1.00)	51	3.75 (1.16)	.04
Perceived usefulness^c^	74	3.84 (0.82)	51	3.49 (0.97)	.03
Perceived nonintrusiveness^c^	74	4.42 (0.65)	51	4.09 (1.03)	.05
Perceived privacy/security^c^	74	4.42 (0.91)	51	3.81 (1.22)	.003
Long-term perceived usefulness	74	4.07 (0.91)	51	3.25 (0.91)	<.001

^a^Values are numbers (column percentages) for categorical variables and mean (SD) for continuous variables.

^b^Two-sample *t* test

^c^Patients’ response at 6 or 12 months. If patients provided responses at 6 and 12 months, then the average of these was used.

When we compared patients who reported a high versus a low willingness to use ATA calls at 18 months, we found six factors to be significantly associated with patients’ reported willingness to use ATA calls. Patients with a high willingness to use ATA calls at 18 months (1) had better diabetes self-care (*P*=.03) and (2) reported a higher willingness to use ATA calls while in the study (*P*=.04); they also reported (3) higher perceived usefulness (*P*=.03), (4) nonintrusiveness (*P*=.05), and (5) privacy/security (*P*=.003) while in the study. Moreover, patients who reported a high willingness to use ATA calls at 18 months also reported (6) higher long-term perceived usefulness (*P*<.001). We jointly entered the six factors into a logistic regression model with backward selection to identify predictive factors. The results revealed that two factors jointly predicted willingness to use ATA calls at 18 months: perceived privacy/security (odds ratio OR=1.59, *P*=.017, 95% CI [1.09, 2.33]) and long-term perceived usefulness (OR=2.77, *P*<.001, 95% CI [1.65, 4.63]).

## Discussion

### Principal Findings

The promises of connected health to efficiently improve access and quality of care [2], rest upon the assumption that patients will readily accept the technologies. Our study on safety-net patient acceptance of automated depression screening and monitoring using ATA calls has important findings suggesting that assumption may be questionable. In the first 6 months of the trial, most patients were accepting of ATA calls and perceived the calls to be private/secure, easy to use, useful, and nonintrusive. Over time, however, patients’ acceptance and their positive perception of ATA call characteristics decreased—although call completion rates remained high and steady. One explanation may be that since ATA call results and prompts for follow-up were sent to DMP care teams during the first 6 months of the trial and to PCPs thereafter, timely follow-up by the latter might have been challenging due to their busy practice loads. Thus, although patients continued to complete ATA calls in the second half of the trial, their PCPs may not have responded to their needs in a timely manner thereby leading them to doubt the value of ATA calls. Furthermore, patients’ acceptance and their perception of ATA call characteristics may also reflect an improvement in their depressive symptoms over time. That is, patients with improved depressive symptoms—due, possibly, to the intervention itself—may no longer perceive the benefits of the ATA calls. We investigated this hypothesis and found that there was no statistically significant difference in the percentage of patients reporting high usefulness and high willingness to use ATA calls among those with improved symptoms, no change in symptoms, or worse symptoms. It may be, however, that our sample size was not large enough to detect these differences.

Another important finding in our study was the identification of two factors that significantly predicted patients’ long-term acceptance of ATA calls: the perception that ATA calls are private/secure and the long-term perceived usefulness of ATA calls. These two factors could be potentially modified to improve patients’ willingness to use ATA calls as part of their depression care.

### Limitations

This study has limitations worth noting. First, we used two different samples for the analyses. For the sample used to determine patient acceptance of ATA calls over time, we included only those patients in the DCAT TC arm who responded to ATA-related measures at each of the corresponding measurement periods. For the sample used to identify factors that predict long-term patient acceptance of ATA calls, we included only those patients in the TC arm who answered the question on willingness to use ATA calls at 18 months and at least once at 6 or 12 months. We chose to accommodate two sample sizes for our study in order to maximize the sample sizes for both analyses, although this may have introduced additional bias.

Second, although the two different samples for the analyses were not significantly different from each other, they were both somewhat different from the rest of TC patients who were excluded from the analyses because they did not answer any of the ATA-related questions. Samples used in the analyses reported a slightly higher willingness to use ATA calls at 6 and 12 months than TC patients excluded from the analyses. However, it is not likely that this limitation affected our findings because only a small percentage (about 10%) of patients excluded from the analyses refused to engage with ATA calls. Nearly 90% of them reported that they could not answer the ATA-related questions because they did not receive or did not remember receiving ATA calls, or they received calls but did not answer because they were unavailable.

Furthermore, the small sample size of 125 patients reporting on willingness to use ATA calls limits the robustness of our findings of factors predicting long-term patient acceptance of ATA calls. Future studies should validate the generalizability of our findings.

A final limitation is that in the analysis of factors predicting long-term acceptance, we defined acceptance as patients’ self-reported willingness to use ATA calls at 18 months instead of using a more objective measure such as ATA call completion rate. This may seem to be a better indicator of patient acceptance, but since we were interested in learning about patients’ *long-term* acceptance, we did not have the ATA call completion rate at 18 months (the intervention was only for 12 months). Moreover, in our qualitative study of DCAT TC patients with incomplete ATA calls, we discovered that patients were actually willing to take the ATA calls, but were unable to do so mainly because of nonintervention related reasons, including not being available, the ATA system having the wrong phone number, or experiencing connection issues [41]. For this reason, we assumed that if patients did not complete ATA calls, it was not due to a lack of acceptance. Therefore, given the DCAT study design and the practical reasons for patients not answering ATA calls, we chose to follow the Patient Technology Acceptance Model (PTAM) [39] and define acceptance as self-reported willingness (ie, intention) to use the technology.

### Comparison With Prior Work

The finding that patients are generally accepting of ATA calls, albeit in the short term, is a promising start to our understanding of patients’ perception of such technologies. Because automated depression screening and monitoring technology is emerging, little is known about patients’ acceptance of it. Related studies of connected health technologies [42], including those focused on depression care [37,43-54], uncritically regard acceptance as patient satisfaction with care, which tells us little about why patients accept or reject the technology or how system design features affect patient acceptance. This study is significant in the connected health literature for depression care in that it utilizes measures from the literature of user acceptance of new technologies [11,55,56]. These user acceptance measures allow us to derive new knowledge that helps not only to explain why the ATA system is acceptable or not to patients, but also to understand how we may improve patient acceptance through the design of the system.

Numerous studies on connected health applications have reported a drop in technology usage over time [57-67]. Unlike these studies, we found that patients’ completion of ATA calls was high and constant throughout the trial. As mentioned above, the main reasons patients reported for incomplete ATA calls were not related to the intervention [41]. In fact, we found in an analysis not included in this paper that the survey-based measures of acceptance were not statistically significant predictors of ATA call completion rates. Nonetheless, as reported in the Results section, the ATA call completion rate was positively correlated with perceived ease of use and perceived nonintrusiveness. The significance of the former factor is in agreement with the PTAM. However, the finding that patients continued to complete ATA calls over time despite a general decrease in acceptance is surprising. Future research is needed to determine whether it was the special characteristics of the study population (ie, predominantly urban, low-income Hispanics/Latinos) or the technology design (ie, outbound calls to patients) that resulted in this finding.

The PTAM sheds light on factors that increase the likelihood that patients will be willing to use connected health technologies. Among a myriad of potential factors, the main ones predicting patient acceptance are perceived usefulness, perceived ease of use, subjective norm, and health care knowledge. Others have similarly reported that perceived usefulness and perceived ease of use are the main driving forces of patient technology acceptance [11,68,69]. Likewise, we found that long-term perceived usefulness of ATA calls significantly predicted patient acceptance of automated depression screening and monitoring. A new predictor of acceptance suggested in our analysis was patients’ perception that calls were private/secure. Future patient technology acceptance research should consider this factor in the technology design and should validate the finding.

### Conclusions

In the short term, safety-net ambulatory care patients with diabetes are generally accepting of ATA calls for depression screening and monitoring. How patient acceptance can be sustained over time is an important topic for future investigation. In order to increase the odds that patients will accept ATA calls over the long term, especially for sensitive mental health conditions, the system should gauge patient perception of its privacy/security. Moreover, it is critically important that the technology not only be aligned with patients’ needs, but also be perceived as useful for them over the long term. Based on the items measuring long-term usefulness, future research should focus on designing and testing technologies that help patients be more aware of how they are feeling, remind them to take care of their health, and help them stay better connected with their health care providers.

## Abbreviations

ATA: automated telephonic assessment
BMI: body mass index
CMS: Centers for Medicare and Medicaid Services
DCAT: Diabetes-Depression Care-management Adoption Trial
DMP: disease management program
DMR: disease management registry
HIT: health information technology
ICD-9: International Classification of Diseases, 9th Revision
LACDHS: Los Angeles County Department of Health Services
PCP: primary care provider
PHQ: Patient Health Questionnaire
SC: supported care
SCL-20: Symptom Checklist, 20 items
SF-12: Short-form Health Survey, 12 items
TC: technology-facilitated care
UC: usual care

## Appendix

Multimedia Appendix 1 DCAT TC Data.

## References

[1] Medicare Program (2014). Office of the Federal Register.

[2] Kvedar J, Coye MJ, Everett W (2014). Connected health: a review of technologies and strategies to improve patient care with telemedicine and telehealth. Health Aff (Millwood).

[3] Dang S, Dimmick S, Kelkar G (2009). Evaluating the evidence base for the use of home telehealth remote monitoring in elderly with heart failure. Telemed J E Health.

[4] Antonicelli R, Testarmata P, Spazzafumo L, Gagliardi C, Bilo G, Valentini M, Olivieri F, Parati G (2008). Impact of telemonitoring at home on the management of elderly patients with congestive heart failure. J Telemed Telecare.

[5] Polisena J, Tran K, Cimon K, Hutton B, McGill S, Palmer K, Scott RE (2010). Home telemonitoring for congestive heart failure: a systematic review and meta-analysis. J Telemed Telecare.

[6] Clark RA, Inglis SC, McAlister FA, Stewart S, Cleland John G F (2007). Telemonitoring or structured telephone support programmes for patients with chronic heart failure: systematic review and meta-analysis. BMJ.

[7] Kulshreshtha Ambar, Kvedar Joseph C, Goyal Abhinav, Halpern Elkan F, Watson Alice J (2010). Use of Remote Monitoring to Improve Outcomes in Patients with Heart Failure: A Pilot Trial. International Journal of Telemedicine and Applications.

[8] Darkins A, Ryan P, Kobb R, Foster L, Edmonson E, Wakefield B, Lancaster AE (2008). Care Coordination/Home Telehealth: the systematic implementation of health informatics, home telehealth, and disease management to support the care of veteran patients with chronic conditions. Telemed J E Health.

[9] Venkatesh V, Morris M, Davis G, Davis F (2003). MIS Quart.

[10] Taylor S, Todd PA (1995). Understanding Information Technology Usage: A Test of Competing Models. Information Systems Research.

[11] Davis FD (1993). User acceptance of information technology: system characteristics, user perceptions and behavioral impacts. International Journal of Man-Machine Studies.

[12] Piette JD (2007). Interactive behavior change technology to support diabetes self-management: where do we stand?. Diabetes Care.

[13] Wu S, Ell K, Gross-Schulman SG, Sklaroff LM, Katon WJ, Nezu AM, Lee P, Vidyanti I, Chou C, Guterman JJ (2014). Technology-facilitated depression care management among predominantly Latino diabetes patients within a public safety net care system: comparative effectiveness trial design. Contemp Clin Trials.

[14] Wu S, Vidyanti I, Liu P, Hawkins C, Ramirez M, Guterman J, Gross-Schulman S, Sklaroff LM, Ell K (2014). Patient-centered technological assessment and monitoring of depression for low-income patients. J Ambul Care Manage.

[15] Alegría Margarita, Chatterji Pinka, Wells Kenneth, Cao Zhun, Chen Chih-nan, Takeuchi David, Jackson James, Meng Xiao-Li (2008). Disparity in depression treatment among racial and ethnic minority populations in the United States. Psychiatr Serv.

[16] Sclar DA, Robison LM, Skaer TL (2008). Ethnicity/race and the diagnosis of depression and use of antidepressants by adults in the United States. Int Clin Psychopharmacol.

[17] Simpson SM, Krishnan LL, Kunik ME, Ruiz P (2007). Racial disparities in diagnosis and treatment of depression: a literature review. Psychiatr Q.

[18] Areán PA, Unützer J (2003). Inequities in depression management in low-income, minority, and old-old adults: a matter of access to preferred treatments?. J Am Geriatr Soc.

[19] Stockdale SE, Lagomasino IT, Siddique J, McGuire T, Miranda J (2008). Racial and ethnic disparities in detection and treatment of depression and anxiety among psychiatric and primary health care visits, 1995-2005. Med Care.

[20] Sánchez-Lacay JA, Lewis-Fernández R, Goetz D, Blanco C, Salmán E, Davies S, Liebowitz M (2001). Open trial of nefazodone among Hispanics with major depression: efficacy, tolerability, and adherence issues. Depress Anxiety.

[21] Heithoff K (1995). Does the ECA underestimate the prevalence of late-life depression?. J Am Geriatr Soc.

[22] Lyness JM, Cox C, Curry J, Conwell Y, King DA, Caine ED (1995). Older age and the underreporting of depressive symptoms. J Am Geriatr Soc.

[23] Sirey JA, Bruce ML, Alexopoulos GS, Perlick DA, Raue P, Friedman SJ, Meyers BS (2001). Perceived stigma as a predictor of treatment discontinuation in young and older outpatients with depression. Am J Psychiatry.

[24] Ell K, Katon W, Xie B, Lee P, Kapetanovic S, Guterman J, Chou C (2010). Collaborative care management of major depression among low-income, predominantly Hispanic subjects with diabetes: a randomized controlled trial. Diabetes Care.

[25] Cabassa LJ, Hansen MC, Palinkas LA, Ell K (2008). Azúcar y nervios: explanatory models and treatment experiences of Hispanics with diabetes and depression. Soc Sci Med.

[26] Huang ES, Zhang JX, Kirchhoff AC, Schaefer CT, Casalino LP, Chin MH, Brown Sydney E S (2008). The cost consequences of improving diabetes care: the community health center experience. Jt Comm J Qual Patient Saf.

[27] Dwight-Johnson M, Unutzer J, Sherbourne C, Tang L, Wells KB (2001). Can quality improvement programs for depression in primary care address patient preferences for treatment?. Med Care.

[28] Miranda J, Duan N, Sherbourne C, Schoenbaum M, Lagomasino I, Jackson-Triche M, Wells KB (2003). Improving care for minorities: can quality improvement interventions improve care and outcomes for depressed minorities? Results of a randomized, controlled trial. Health Serv Res.

[29] Oxman TE, Dietrich AJ, Williams JW, Kroenke K (2002). A three-component model for reengineering systems for the treatment of depression in primary care. Psychosomatics.

[30] Oxman TE, Dietrich AJ, Schulberg HC (2003). The depression care manager and mental health specialist as collaborators within primary care. Am J Geriatr Psychiatry.

[31] Badamgarav E, Weingarten SR, Henning JM, Knight K, Hasselblad V, Gano A, Ofman JJ (2003). Effectiveness of disease management programs in depression: a systematic review. Am J Psychiatry.

[32] Henke RM, McGuire TG, Zaslavsky AM, Ford DE, Meredith LS, Arbelaez JJ (2008). Clinician- and organization-level factors in the adoption of evidence-based care for depression in primary care. Health Care Manage Rev.

[33] Post EP, Kilbourne AM, Bremer RW, Solano FX, Pincus HA, Reynolds CF (2009). Organizational factors and depression management in community-based primary care settings. Implement Sci.

[34] Nutting PA, Gallagher K, Riley K, White S, Dickinson WP, Korsen N, Dietrich A (2008). Care management for depression in primary care practice: findings from the RESPECT-Depression trial. Ann Fam Med.

[35] U.S. Preventive Services Task Force (2009). Screening for depression in adults: U.S. preventive services task force recommendation statement. Ann Intern Med.

[36] Bodenheimer T, Wagner EH, Grumbach K (2002). Improving primary care for patients with chronic illness: the chronic care model, Part 2. JAMA.

[37] Kroenke K, Theobald D, Wu J, Norton K, Morrison G, Carpenter J, Tu W (2010). Effect of telecare management on pain and depression in patients with cancer: a randomized trial. JAMA.

[38] Hu P, Chau P, Sheng O, Tam K (1999). Examining the Technology Acceptance Model Using Physician Acceptance of Telemedicine Technology. Journal of Management Information Systems.

[39] Karsh B, Severtson DJ, Burke LJ, Brown RL, Brennan PF, Or Calvin K L (2011). Factors affecting home care patients' acceptance of a web-based interactive self-management technology. J Am Med Inform Assoc.

[40] Legris P, Ingham J, Collerette P (2003). Why do people use information technology? A critical review of the technology acceptance model. Information & Management.

[41] Vidyanti I, Wu B, Wu S (2015). Low-income minority patient engagement with automated telephonic depression assessment and impact on health outcomes. Qual Life Res.

[42] Williams TL, May CR, Esmail A (2001). Limitations of patient satisfaction studies in telehealthcare: a systematic review of the literature. Telemed J E Health.

[43] Clarke G, Reid E, Eubanks D, O'Connor E, DeBar LL, Kelleher C, Lynch F, Nunley S (2002). Overcoming depression on the Internet (ODIN): a randomized controlled trial of an Internet depression skills intervention program. J Med Internet Res.

[44] Christensen H, Griffiths KM, Jorm AF (2004). Delivering interventions for depression by using the internet: randomised controlled trial. BMJ.

[45] Andersson G, Bergström J, Holländare F, Carlbring P, Kaldo V, Ekselius L (2005). Internet-based self-help for depression: randomised controlled trial. Br J Psychiatry.

[46] Lynch DJ, Tamburrino MB, Nagel R (1997). Telephone counseling for patients with minor depression: preliminary findings in a family practice setting. J Fam Pract.

[47] Hunkeler EM, Meresman JF, Hargreaves WA, Fireman B, Berman WH, Kirsch AJ, Groebe J, Hurt SW, Braden P, Getzell M, Feigenbaum PA, Peng T, Salzer M (2000). Efficacy of nurse telehealth care and peer support in augmenting treatment of depression in primary care. Arch Fam Med.

[48] Mohr DC, Likosky W, Bertagnolli A, Goodkin DE, Dwyer P, Dick LP, Van Der Wende J (2000). Telephone-administered cognitive-behavioral therapy for the treatment of depressive symptoms in multiple sclerosis. J Consult Clin Psychol.

[49] Miller L, Weissman M (2002). Interpersonal psychotherapy delivered over the telephone to recurrent depressives. A pilot study. Depress Anxiety.

[50] Lynch D, Tamburrino M, Nagel R, Smith MK (2004). Telephone-based treatment for family practice patients with mild depression. Psychol Rep.

[51] Lieberman MA, Winzelberg A, Golant M, Wakahiro M, DiMinno M, Aminoff M, Christine C (2005). Online support groups for Parkinson's patients: a pilot study of effectiveness. Soc Work Health Care.

[52] Spek V, Nyklícek I, Smits N, Cuijpers P, Riper H, Keyzer J, Pop V (2007). Internet-based cognitive behavioural therapy for subthreshold depression in people over 50 years old: a randomized controlled clinical trial. Psychol Med.

[53] Warmerdam L, van SA, Twisk J, Riper H, Cuijpers P (2008). Internet-based treatment for adults with depressive symptoms: randomized controlled trial. J Med Internet Res.

[54] Meyer B, Berger T, Caspar F, Beevers CG, Andersson G, Weiss M (2009). Effectiveness of a novel integrative online treatment for depression (Deprexis): randomized controlled trial. J Med Internet Res.

[55] Davis FD (1989). Perceived Usefulness, Perceived Ease of Use, and User Acceptance of Information Technology. MIS Quarterly.

[56] Dillon A, Morris M (1996). User acceptance of new information technology: theories and models. Annu Rev Inform Sci Technol.

[57] Glasgow RE, Boles SM, McKay HG, Feil EG, Barrera M (2003). The D-Net diabetes self-management program: long-term implementation, outcomes, and generalization results. Prev Med.

[58] Lorig KR, Ritter PL, Laurent DD, Plant K (2006). Internet-based chronic disease self-management: a randomized trial. Med Care.

[59] Heisler M, Piette JD (2005). “I help you, and you help me”: facilitated telephone peer support among patients with diabetes. Diabetes Educ.

[60] Fisher L, Chesla CA, Skaff MM, Gilliss C, Mullan JT, Bartz RJ, Kanter RA, Lutz CP (2000). The family and disease management in Hispanic and European-American patients with type 2 diabetes. Diabetes Care.

[61] Balas EA, Krishna S, Kretschmer RA, Cheek TR, Lobach DF, Boren SA (2004). Computerized knowledge management in diabetes care. Med Care.

[62] Murray E, Burns J, See TS, Lai R, Nazareth I (2005). Interactive Health Communication Applications for people with chronic disease. Cochrane Database Syst Rev.

[63] Young AS, Chaney E, Shoai R, Bonner L, Cohen AN, Doebbeling B, Dorr D, Goldstein MK, Kerr E, Nichol P, Perrin R (2007). Information technology to support improved care for chronic illness. J Gen Intern Med.

[64] Dorr D, Bonner LM, Cohen AN, Shoai RS, Perrin R, Chaney E, Young AS (2007). Informatics systems to promote improved care for chronic illness: a literature review. J Am Med Inform Assoc.

[65] Tate DF, Jackvony EH, Wing RR (2003). Effects of Internet behavioral counseling on weight loss in adults at risk for type 2 diabetes: a randomized trial. JAMA.

[66] Tate DF, Wing RR, Winett RA (2001). Using Internet technology to deliver a behavioral weight loss program. JAMA.

[67] Glasgow RE, Toobert DJ, Brown J, Hampson SE, Riddle MC, La Chance P A (1997). Long-term effects and costs of brief behavioural dietary intervention for patients with diabetes delivered from the medical office. Patient Educ Couns.

[68] Jimison H, Gorman P, Woods S, Nygren P, Walker M, Norris S, Hersh W (2008). Barriers and drivers of health information technology use for the elderly, chronically ill, and underserved. Evid Rep Technol Assess (Full Rep).

[69] Davis FD, Bagozzi RP, Warshaw PR (1989). User Acceptance of Computer Technology: A Comparison of Two Theoretical Models. Management Science.

