# Supplementary material for: Automated Remote Monitoring of Depression: Acceptance Among Low-Income Patients in Diabetes Disease Management
Source: JMIR Ment Health. 2016 Jan 25;3(1):e6. doi: 10.2196/mental.4823 (PMC4736285; doi:10.2196/mental.4823)
Supplement: Multimedia Appendix 1 [file mental_v3i1e6_app1.pdf]

Multimedia Appendix: DCAT TC Data

Table A-1. Comparison of samples in the first analysis to the rest of patients in the TC arm of DCAT

Characteristic	Sample for First Analysis		Rest of Patients in the TC Arm of DCAT		P[b]
	N	Statistics[a]	N	Statistics[a]	
Female	109	72 (66.1%)	333	201 (60.4%)	0.29
Age	109	51.94 (9.01)	333	52.80 (8.87)	0.38
Hispanic/Latino	109	105 (96.3%)	332	295 (88.9%)	0.02
Spanish as preferred language	109	93 (85.0%)	333	268 (80.5%)	0.26
Married	109	49 (45.0%)	333	174 (52.3%)	0.19
PHQ-9 (range 0–27, higher=more severe depression)[b, c]	109	5.73 (4.93)	333	5.48 (5.05)	0.66
Total number of socioeconomic stressors[c]	109	2.28 (1.56)	333	2.35 (1.59)	0.72
SCL-20, mean score[c, d]	109	0.54 (0.53)	333	0.51 (0.52)	0.62
SF-12 mental (general population=50, higher=better)[c, e]	109	50.54 (9.15)	333	50.96 (10.09)	0.70
Time with diabetes in years	107	10.15 (7.42)	330	10.61 (7.14)	0.57
On insulin treatment[c]	109	82 (75.2%)	333	229 (68.8%)	0.20
BMI[c, f]	109	32.93 (6.55)	333	32.93 (6.81)	1.00
A1C value[c, g]	108	8.87 (1.39)	332	9.12 (1.60)	0.14
Low-density lipoprotein cholesterol[c,]	108	167.08 (36.20)	332	171.82 (39.51)	0.27
Whitty-9 diabetes symptoms (range 1–5, 1=none to 5=every day)[c]	109	1.64 (0.54)	333	1.60 (0.49)	0.45
Number of diabetes complications[c]	109	1.26 (0.89)	333	1.26 (0.97)	0.96
Toolbert diabetes self-care in the past 7 days (range 0–7)[c]	109	4.63 (0.98)	333	4.42 (1.08)	0.08
Diabetes emotional burden (range 1–5, 1=not a problem to 5=very burdensome)[c]	109	2.53 (1.35)	333	2.49 (1.50)	0.79
Diabetes regime distress (range 1–5, 1=not a problem to 5=very burdensome)[c]	109	2.19 (1.14)	333	2.19 (1.37)	0.98
Self-rated health (range 1–5, 1=poor to 5=excellent)[c]	109	2.29 (0.60)	333	2.37 (0.71)	0.25
Chronic pain[c]	109	17 (15.6%)	333	58 (17.4%)	0.66
SF-12 physical (general	109	43.18 (9.62)	333	43.85 (9.50)	0.52

Characteristic	Sample for First Analysis		Rest of Patients in the TC Arm of DCAT		P^b
	N	Statistics[a]	N	Statistics[a]	
population=50, higher=better health)[c, e]					
Sheehan disability scale (range 0–10, 0=none to 10=extremely)[c]	109	2.21 (2.34)	333	2.14 (2.43)	0.81
Number of ICD-9 diagnosis[c, h]	108	8.60 (4.50)	332	7.96 (4.14)	0.18
Number of clinic visits[c]	107	10.44 (5.61)	317	9.54 (5.56)	0.15
Number of emergency room visits[c]	41	1.33 (0.61)	90	1.23 (0.42)	0.35
Number of hospitalizations[c]	15	1.47 (0.83)	33	1.09 (0.26)	0.10
Willingness to use[c]	109	4.02 (0.93)	153	3.56 (1.40)	0.002
Perceived ease-of-use[c]	109	4.05 (0.56)	146	3.98 (0.66)	0.36
Perceived usefulness[c]	109	3.63 (0.89)	145	3.60 (0.94)	0.81
Perceived non-intrusiveness[c]	109	4.20 (0.87)	146	4.02 (1.05)	0.13
Perceived privacy/security[c]	109	4.10 (1.11)	148	4.14 (1.12)	0.81
Preference of ATA call mode[c]	109	3.82 (1.06)	152	3.42 (1.53)	0.01
Long-term perceived usefulness	76	3.71 (0.92)	71	3.68 (1.13)	0.84
ATA call completion rate[c]	108	0.70 (0.26)	303	0.50 (0.33)	<.001

[a]Values are numbers (column percentages) for categorical variables and mean (SD) for continuous variables
[b]Chi-square test for categorical variables and two-sample t-test for continuous variables
[c]Assessment at 6 or 12 months. If both were available, then the average was taken.
[d]Symptoms CheckList, 20 items
[e]Short-Form Health Survey, 12 items
[f]Body mass index
[g]Glycated hemoglobin test
[h]International Classification of Diseases, 9th Revision

Table A-2. Comparison of samples in the second analysis to the rest of patients in the TC arm of DCAT

Characteristic	Sample for Second Analysis		Rest of Patients in the TC Arm of DCAT		P^b
	N	Statistics[a]	N	Statistics[a]	
Female	125	80 (64.0%)	333	201 (60.4%)	0.29
Age	125	51.31 (8.81)	317	53.09 (8.91)	0.06
Hispanic/Latino	125	116 (92.8%)	332	295 (88.9%)	0.02
Spanish as preferred language	125	104 (83.2%)	333	268 (80.5%)	0.26
Married	125	55 (44.0%)	333	174 (52.3%)	0.19
PHQ-9 (range 0–27,	125	5.65 (4.60)	317	5.50 (5.18)	0.78

Characteristic	Sample for Second Analysis		Rest of Patients in the TC Arm of DCAT		*P*[b]
	N	Statistics[a]	N	Statistics[a]	
higher=more severe depression)[b, c]					
Total number of socioeconomic stressors[c]	125	2.37 (1.46)	317	2.31 (1.63)	0.72
SCL-20, mean score[c, d]	125	0.51 (0.48)	317	0.52 (0.54)	0.89
SF-12 mental (general population=50, higher=better)[c, e]	125	51.08 (9.03)	317	50.76 (10.18)	0.76
Time with diabetes in years	124	9.98 (7.05)	313	10.70 (7.27)	0.35
On insulin treatment[c]	125	89 (71.2%)	317	222 (70.0%)	0.81
BMI[c, f]	125	32.75 (6.16)	317	33.00 (6.97)	0.73
A1C value[c, g]	124	8.72 (1.39)	316	9.20 (1.59)	0.003
Low-density lipoprotein cholesterol[c]	124	168.44 (36.60)	316	171.53 (39.57)	0.45
Whitty-9 diabetes symptoms (range 1–5, 1=none to 5=every day)[c]	125	1.62 (0.49)	317	1.60 (0.50)	0.65
Number of diabetes complications[c]	125	1.22 (0.79)	317	1.27 (1.01)	0.57
Toolbert diabetes self-care in the past 7 days (range 0–7)[c]	125	4.65 (1.01)	317	4.40 (1.07)	0.02
Diabetes emotional burden (range 1–5, 1=not a problem to 5=very burdensome)[c]	125	2.48 (1.37)	317	2.50 (1.50)	0.90
Diabetes regime distress (range 1–5, 1=not a problem to 5=very burdensome)[c]	125	2.13 (1.17)	317	2.21 (1.37)	0.58
Self-rated health (range 1–5, 1=poor to 5=excellent)[c]	125	2.34 (0.60)	317	2.36 (0.71)	0.81
Chronic pain[c]	125	24 (19.2%)	333	51 (16.1%)	0.43
SF-12 physical (general population=50, higher=better health)[c, e]	125	43.17 (9.49)	317	43.88 (9.54)	0.48
Sheehan disability scale (range 0–10, 0=none to 10=extremely)[c]	125	2.14 (2.26)	317	2.17 (2.46)	0.91
Number of ICD-9 diagnosis[c, h]	124	8.46 (4.46)	316	7.98 (4.14)	0.29
Number of clinic visits[c]	124	10.56 (5.64)	300	9.44 (5.53)	0.06
Number of emergency room visits[c]	44	1.33 (0.60)	87	1.09 (0.27)	0.31
Number of hospitalizations[c]	18	1.39 (0.78)	30	1.09 (0.27)	0.14
Willingness to use[c]	125	4.00 (1.08)	137	3.53 (1.34)	0.002
Perceived ease-of-use[c]	125	4.12 (0.50)	130	3.91 (0.70)	0.005

Characteristic	Sample for Second Analysis		Rest of Patients in the TC Arm of DCAT		*P*[b]
	N	Statistics[a]	N	Statistics[a]	
Perceived usefulness[c]	125	3.69 (0.90)	129	3.54 (0.93)	0.17
Perceived non-intrusiveness[c]	125	4.29 (0.84)	130	3.92 (0.70)	0.003
Perceived privacy/security[c]	125	4.17 (1.08)	132	4.07 (1.14)	0.47
Preference of ATA call mode[c]	125	3.58 (1.32)	136	3.59 (1.41)	0.98
Long-term perceived usefulness	125	3.74 (0.99)	22	3.45 (1.18)	0.24
ATA call completion rate[c]	123	0.74 (0.24)	288	0.48 (0.32)	<.001

[a]Values are numbers (column percentages) for categorical variables and mean (SD) for continuous variables
[b]Chi-square test for categorical variables and two-sample t-test for continuous variables
[c]Assessment at 6 or 12 months. If both were available, then the average was taken.
[d]Symptoms CheckList, 20 items
[e]Short-Form Health Survey, 12 items
[f]Body mass index
[g]Glycated hemoglobin test
[h]International Classification of Diseases, 9th Revision

Table A-3. Characteristics of patients reporting high versus low willingness to use ATA calls at 18 months

Characteristic	High willingness to use ATA calls at 18 months		Low willingness to use ATA calls at 18 months		*P*[b]
	N	Statistics[a]	N	Statistics[a]	
Female	74	52 (70.3%)	51	28 (54.9%)	0.08
Age	74	51.97 (8.56)	51	50.35 (9.16)	0.41
Hispanic/Latino	74	71 (95.9%)	51	45 (88.2%)	0.10
Spanish as preferred language	74	65 (87.8%)	51	39 (76.5%)	0.10
Married	74	32 (43.2%)	51	23 (45.1%)	0.84
PHQ-9 (range 0–27, higher=more severe depression)[b, c]	74	5.62 (4.58)	51	5.69 (4.69)	0.94
Total number of socioeconomic stressors[c]	74	2.46 (1.45)	51	2.25 (1.48)	0.38
SCL-20, mean score[c, d]	74	0.52 (0.47)	51	0.50 (0.51)	0.84
SF-12 mental (general population=50, higher=better)[c, e]	74	50.40 (9.18)	51	52.07 (8.81)	0.31
Time with diabetes in years	73	9.88 (7.12)	51	10.14 (7.01)	0.84
On insulin treatment[c]	74	53 (71.6%)	51	36 (70.6%)	0.90
BMI[c, f]	74	32.54 (6.20)	51	33.06 (6.16)	0.65
A1C value[c, g]	74	8.71 (1.30)	50	8.73 (1.53)	0.91
Low-density lipoprotein	74	172.03	50	166.10	0.56

Characteristic	High willingness to use ATA calls at 18 months		Low willingness to use ATA calls at 18 months		P^b
	N	Statistics[a]	N	Statistics[a]	
cholesterol[c]		(37.36)		(35.68)	
Whitty-9 diabetes symptoms (range 1–5, 1=none to 5=every day)[c]	74	1.59 (0.46)	51	1.67 (0.53)	0.34
Number of diabetes complications[c]	74	1.27 (0.83)	51	1.15 (0.73)	0.42
Toolbert diabetes self-care in the past 7 days (range 0–7)[c]	74	4.81 (0.95)	51	4.43 (1.05)	0.03
Diabetes emotional burden (range 1–5, 1=not a problem to 5=very burdensome)[c]	74	2.50 (1.37)	51	2.46 (1.37)	0.87
Diabetes regime distress (range 1–5, 1=not a problem to 5=very burdensome)[c]	74	2.14 (1.14)	51	2.13 (1.23)	0.96
Self-rated health (range 1–5, 1=poor to 5=excellent)[c]	74	2.37 (0.59)	51	2.30 (0.62)	0.52
Chronic pain[c]	74	13 (17.6%)	51	11 (21.6%)	0.58
SF-12 physical (general population=50, higher=better health)[c, e]	74	43.48 (9.53)	51	42.72 (9.51)	0.66
Sheehan disability scale (range 0–10, 0=none to 10=extremely)[c]	74	2.13 (2.08)	51	2.15 (2.53)	0.97
Number of ICD-9 diagnosis[c, h]	74	8.30 (4.02)	50	8.69 (5.08)	0.63
Number of clinic visits[c]	74	10.97 (5.75)	50	9.96 (5.47)	0.33
Number of emergency room visits[c]	29	1.44 (0.69)	15	1.10 (0.28)	0.07
Number of hospitalizations[c]	11	1.45 (0.82)	7	1.29 (0.76)	0.67
Willingness to use[c]	74	4.17 (1.00)	51	3.75 (1.16)	0.04
Perceived ease-of-use[c]	74	4.17 (0.50)	51	4.05 (0.51)	0.17
Perceived usefulness[c]	74	3.84 (0.82)	51	3.49 (0.97)	0.03
Perceived non-intrusiveness[c]	74	4.42 (0.65)	51	4.09 (1.03)	0.05
Perceived privacy/security[c]	74	4.42 (0.91)	51	3.81 (1.22)	0.003
Preference of ATA call mode[c]	74	3.66 (1.35)	51	3.47 (1.29)	0.43
Long-term perceived usefulness	74	4.07 (0.91)	51	3.25 (0.91)	<.001
ATA call completion rate[c]	72	0.75 (0.23)	51	0.73 (0.25)	0.56

[a]Values are numbers (column percentages) for categorical variables and mean (SD) for continuous variables

[b]Chi-square test for categorical variables and two-sample t-test for continuous variables

[c]Assessment at 6 or 12 months. If both were available, then the average was taken.

[d]Symptoms CheckList, 20 items

[e]Short-Form Health Survey, 12 items
[f]Body mass index
[g]Glycated hemoglobin test
[h]International Classification of Diseases, 9th Revision
